# The Internal Use of Carbolic Acid

**Published:** 1869-10

**Authors:** Joseph G. Pinkham

**Affiliations:** Lynn, Mass.


### Miscellaneous.

### The Internal Use of Carbolic Acid.

BY JOSEPH G. PINKHAM, A. M. M. D., of Lynn, Mass.

Since the first introduction of carbolic acid to the notice of the profession, as a remedial agent, in the early part of the present decade, many pages, I might, perhaps, say volumes, have been written, pro and con, upon the question of its therapeutic merits. And although in some respects its good qualities are pretty thoroughly established, in others it must still be considered as *sub judice*—a statement, forsooth, that might be truthfully made concerning every article of the materia medica, so uncertain is the greater par- of our so-called knowledge, in regard to the action of drugs upon the human system, in health and disease.

The power possessed by carbolic acid, in common with several other allied compounds extracted from coal tar, of arresting the processes of putrefaction and fermentation, and of destroying the germs of organic life, or of preventing their development, is generally considered the chief basis of its therapeutic value; and it was the discovery of this power that prepared the way for its employment externally as a disinfectant, deodorant and preservative. The manifest success of its application for these purposes, led, very naturally, at a time when the minds of medical men were possessed by the notion of the zymotic or fungous origin of certain diseases, to its use internally. Who first thought favorably enough of the idea to put it into actual practice, it would be difficult now to ascertain. Probably the credit of originality is due equally to more than one observer. The editor of Braithwaite seems to ascribe it to Dr.

Keith, of Normandy, England,* but in this he is widely mistaken, as will appear by the citations further on.

When administered internally, whether by the stomach, in the form of spray by inhalation, or as an injection to be retained, the remedy is always applied to a mucous surface, and from it absorbed into the blood. Hence there are three classes of effects to be studied. 1. The local effects upon the mucous membrane. 2. Effects upon the substances in contact with the mucous membrane. 3. Effects upon the blood and vital processes after absorption.

The local effects, when employed in very dilute solution, as, of course, it always is in the methods of administration mentioned above, are those of moderate and agreeable stimulation. It has been called a local sedative; and it certainly does allay nervous irritation, itching, &c. This may, however, be mostly a secondary effect of its chemical, or of its stimulant action.

Its effect upon the substances in contact with the membrane, whether mucous, pus, blood, the digestive fluids, aliment, fæces or other matter, are chiefly in the direction of arresting or preventing destructive chemical changes. In this way it prevents the formation of new substances which irritate the mucous membrane, and cause distress, or aggravate any existing derangement of function or condition. Its powers as a local alterative are, I opine, due mainly to this action.

It has been suggested that it might retard the process of digestion, when taken into the stomach with the food, by interfering with the action of the nitrogenized constituents of the digestive fluids. I have tested its influence upon the salivary and gastric fluids with respect to their peculiar action upon the food, in the manner described below.

*Experiment I.*—The fluids of the mouth were tested for sugar, and found to contain none, or only the merest trace. A piece of boiled starch, free from sugar, was held in the mouth for five minutes, filtered, and tested for sugar. A beautiful and decided reaction, showing its presence in large quantity.

*Experiment II.*—A portion of starch was intimately incorporated with an aqueous solution of carbolic acid, and held in the mouth for five minutes. Test, and result as in Exp. I.

*Experiment III.*—A piece of starch intimately mingled with a larger quantity of carbolic acid, as much as could be comfortably borne by the mouth, was subjected to the same process with the same result as in the other two experiments.

These experiments were repeated, with the variation of holding the starch in the mouth a much shorter period, from one half minute to a minute, with exactly the same result, except that the test showed less sugur to be present in each instance.

*Experiment IV.*—An artificial gastric juice was made by macer-

---

* Braithwaite's Retrospect, July, 1869, p. 26.

rating the stomach of a recently killed cat, in an ounce of water, acidulated with thirty minims of hydrochloric acid. This was filtered, and a clear, slightly yellow liquid obtained. Seven test-tubes of the ordinary size were then tightly fitted with corks, and into each one was put forty minims of the "gastric juice," and a small shred of beef. The shreds of beef were all, as nearly as could be judged by the eye, of equal size. To test tubes *a* and *b* no further additions were made. To *c* was added 1-24 minim of carbolic acid dissolved in water. To *d* was added 1-6 minim of carbolic acid dissolved in water. To *e* was added 1-3 minim of carbolic acid dissolved in water. To *f* was added one small drop of carbolic acid, taken up by a glass rod. To *g* were added two such drops. The whole were then placed at a temperature of 96° ad 100° F., agitated occasionally, and the result noted at the end of six hours, as follows:—

Test tubes *a* and *b*, meat completely digested; fine, red sediment at the bottom of the tubes; liquid above somewhat whey-like.

Test tube *c*, meat completely disintegrated; a few minute muscular fibres floating about undisturbed.

Test tube *d*, meat partially disgested; solid fragments still undissolved.

Test tube *e*, meat disgested to a less degree than that in *d*.

Test tube *f*, meat only slightly acted upon, shred remaining entire.

Test tube *g*, same as in *f*.

After the lapse of ten hours the meat in *d* was found completely digested. In eighteen hours that in *e* was nearly digested. In the others there was no further change apparent.

From these experiments, granting that the solution used in the last was a fair equivalent for the gastric juice, and that there has been no error of observation, we may, I think, deduce the following conclusions:—

1. Carbolic acid does not prevent, and probably does not retard, the conversion of starch into sugar by the salivary fluids.

2. When present in no greater proportion than one part in one thousand, carbolic acid does not interfere seriously with the solvent action of the gastric juice upon the nitrogenized constituents of food.

3. When present in larger proportion it interferes with this solvent action, according to its amount. The interference is decided when one part in three hundred and twenty is present; and the one hundred and twentieth part entirely prevents digestion.

From these deductions we may assume that, given in the ordinary doses of the acid, no impairment of primary digestion need be apprehended. When given in doses sufficiently large to retard digestion, the constitutional, poisonous effects will be developed, and overshadow the others. Still it is reasonable to suppose that given with the food for a long time, even in small quantities, it might have an unfavorable effect upon the economy, owing to the power which the experiments prove it to possess.

With reference to its effects upon the blood and vital processes after absorption, a grave question arises. Has it any injurious control over ultimate nutrition—over those processes of waste and repair which are constantly going on in the organism, and the due balance of which is essential to health? This question cannot be answered. It unfortunately belongs to that dark domain of ignorance and conjecture, vital chemistry. We can, however, safely assume that there is a *probability* of its possessing such control; and this probability, together with the conclusions arrived at from the experiments, seem to indicate, very clearly, a limit to the therapeutic usefulness of carbolic acid, when administered internally. But within this limit there is a large class of morbid conditions which, we should naturally conclude, would be favorably affected by its use. And there is not wanting abundant testimony (however valuable or valueless, it may be considered) to its efficacy. I make a few references in order to show the direction in which the testimony tends.

Dr. Godfrey, of England, advises its use internally for gastric irritability, the vomiting of pregnancy, flatulence from imperfect digestion, and certain forms of diarrhœa.*

Dr. Kempton, of Utica, N. Y., has found it of advantage in a somewhat similar class of affections, such as sluggishness of the bowels with offensive breath, dyspepsia with eructations of gas, a yeasty condition of the stomach, diarrhœa from eating unripe fruit, &c. The doses in which he gave it were one or two drachms of a solution of one grain to an ounce of water, pro re nata. He also employed the remedy with success by inhalation for nasal catarrh with profuse, offensive discharges, and by gargle for sore throat in scarlet fever, diphtheria, and simple tonsillitis.§

Dr. Wolfe, of Aberdeen, believes it beneficial in all stages of phthisis, particularly for arresting hæmoptysis, allaying irritation, and arresting the profuse secretion in cases of chronic bronchitis and of cavities in the lungs, of laryngeal-phthisis and of colliquative sweats. †

Mr. Blake, of Birmingham, deems it of very great use in whooping cough, given by inhalation. ‡

Dr. Andrew Clark, at the London Hospital, considers it valuable in the treatment of vomiting associated with fermentation and catarrh, given in one grain doses in pill; in hæmatemesis from gastric erosions, or ulcer, one grain dissolved in water, with a little spirit every two or three hours; in atonic cases of chronic gastric catarrh, where bismuth, silver, and the acids have failed, in quarter grain doses, much diluted, upon an empty stomach, to be preceded by two or three days' employment of bicarbonate of soda, with or without hydrocyanic acid; in water brash, grain doses with

* Medical Circular, Dec. 17th. 1862.

§ American Journal of the Medical Sciences. July, 1868.

† Med. Times and Gazette, Nov. 25. 1865, Braithwaite, Part liii, p. 87.

‡ Med. Times and Gazette, April 11, 1868, opium and bismuth; in flatulence, for temporary relief, in grain doses, in pill; in chronic bronchitis, and bronchorrhœa, taken by the stomach in half grain doses dissolved in water, several times a day, and by inhalation of vapor produced by the addition of twenty drops of deliquesced acid to a pint of boiling water, or in the form of spray by the atomizer, one grain to six ounces of water; in certain forms of phthisis in which there is much secretion from bronchial tubes or cavities, and not much irritation, vapor from boiling water (spray cannot be used with safety in any case of phthisis); in oozing hæmorrhages from air passages, in diarrhœa accompanying the march of epidemic cholera, in mucous disease of the large intestine, given by inhalation.

Dr. Clark thinks the remedy of no use in cholera, and so far as his experience goes, of little value in fevers.* It will be observed that his testimony in regard to its efficacy in phthisis is directly in opposition to that of Dr. Wolfe, on some points.

Dr. Fuller, of London, employs it in six or eight minim doses of the deliquesced acid for dyspeptic cases of the fermentative class; in scarlatina with sloughing throat; and in the form of spray in the early and advanced stages of phthisis, in laryngeal phthisis, chronic bronchitis, gangrene of the lung, and various affections of the throat. Solution five to ten minims to ounce of water.† He does not find that it exerts any controlling influence over typhoid and gastric (?) fevers.

Dr. Garraway places great reliance upon it in the vomiting of pregnancy. He gives drop doses three times a day.‡

Dr. Keith recommends it internally for scarlet fever, smallpox and measles. The therapeutical effects which he attributes to it are profuse perspiration; rapid lowering of pulse; reduction of fever; improvement of tongue and throat; increase of appetite, all after its use for twenty-four hours. He thinks it most useful in the early stages, but given afterwards it very much modifies the symptoms and carries the patient through the different stages more rapidly than any other treatment he has seen. He noticed that in some cases the urine appeared smoky, as if fine charcoal had been used with it.§ He prescribed the remedy in combination with acetic acid, laudanum, and chloric ether. For this reason it is difficult to know how much weight to give to his conclusions. A rigid criticism would certainly reject them altogether.

It is manifestly unreasonable to combine several articles of the materia medica, some of them of known potency, and some of them of unknown, and ascribe all the supposed effects to the unknown. To ascribe them to the medicine at all, may involve the fallacy of *quia post ergo propter*, but the course indicated above, too often, indeed, followed, tends to introduce a new and more alarming ele-

* British Med. Journal, Feb. 13, 1869.

† British Med. Journal. Feb. 20, 1869.

‡ British Med. Journal, March 13, 1869.

§ London Lancet, Jan. 23, 1869. Braithwaite, Part lix. p. 26.

ment of uncertainty into the conclusions. We have too much of imperfect observation and false reasoning in therapeutics. Our periodicals are filled with statements of the efficacy of this or that drug in the treatment of that disease, and yet, as a rule, we distrust them all, for we have learned from experience how unreliable they are. The lines of scientific criticism have not yet been drawn closely enough in this department of medical research.

It will have been observed that the doses in which carbolic acid has been given by different experimenters, varies from one-eighth grain of the crystals up to eight minims of the deliquesced acid, in pill or solution, by the stomach, and from one grain to ten minims to an ounce of water, for inhalation. My own dose ranges from one minim to three, three times a day, or oftener if required, by the stomach, and from one minim to five to the ounce of water for inhalation. I prefer to give it always in largely diluted aqueous solution. The taste can be to some extent masked by a little lemon juice or cinnamon water. Dr. Fuller, who uses the largest doses of any one who has written upon the subject, has noticed faintness follow occasionally the long-continued application of the spray.* I have several times noticed dizziness and faintness following the use of three minim doses taken into the stomach, in dilute solution. I consider that three minims should be the maximum dose. We ought not to lose side of the fact that carbolic acid, like other potent medicines, is a dangerous poison; that it has proved fatal in more than one instance when applied as a wash to the whole surface of the body in some skin diseases; and several times when taken internally.

It would be difficult to state the smallest amount that could prove fatal to an adult I should tremble for the safety of a patient who had taken half a drachm, or even twenty minims, in however weak solution.—*Boston Med. Journal.*

